# A functional polymorphism rs10830963 in *melatonin receptor 1B* associated with the risk of gestational diabetes mellitus

**DOI:** 10.1042/BSR20190744

**Published:** 2019-12-20

**Authors:** Bo Huang, Yu-kun Wang, Lin-yuan Qin, Qin Wei, Nian Liu, Min Jiang, Hong-ping Yu, Xiang-yuan Yu

**Affiliations:** 1 Department of Epidemiology and Health Statistics of Guilin Medical University, Guilin 541100, Guangxi, China; 2 Department of Microbiology of Guangxi University, Nanning 530004, Guangxi, China; 3 Affiliated Tumor Hospital of Guangxi Medical University, Nanning 530021, Guangxi, China

**Keywords:** Gestational diabetes mellitus, meta analysis, MTNR1B, Polymorphism, Trial sequential analysis

## Abstract

*The melatonin receptor 1B* (*MTNR1B*) polymorphism rs10830963 C>G has been reported to be associated with the risk of gestational diabetes mellitus (GDM) with inconsistent results. To clarify the effect of the polymorphism on the risk of GDM, a meta-analysis therefore was performed. Pooled OR with its corresponding 95%CI was used to estimate the strength of the association. Totally 14 eligible studies with a number of 5033 GDM patients and 5614 controls were included in this meta-analysis. Results indicated that the variant G allele was significantly associated with an increased GDM risk (CG vs. CC: OR = 1.25, 95% CI = 1.11−1.40, *P* < 0.001; GG vs. CC: OR = 1.78, 95% CI = 1.45−2.19, *P* < 0.001; G vs. C: OR = 1.33, 95% CI = 1.21−1.47, *P* < 0.001). In the stratified analysis by ethnicity, similar results were found in Asians (CG vs. CC: OR = 1.15, 95%CI = 1.02−1.28, *P* = 0.020; GG vs. CC: OR = 1.52, 95% CI = 1.23−1.89, *P* < 0.001; G vs. C: OR = 1.23, 95% CI = 1.10−1.37, *P* < 0.001) and in Caucasians (CG vs. CC: OR = 1.40, 95% CI = 1.16−1.70, *P* < 0.001; GG vs. CC: OR = 2.21, 95% CI = 1.54−3.17, *P* < 0.001; G vs. C: OR = 1.47, 95% CI = 1.24−1.73, *P* < 0.001). FPRP and TSA analyses confirmed findings support that the rs10830963 G allele increases the risk of GDM, and further functional experimental studies are warranted to explore and clarify the potential mechanism.

## Introduction

Gestational diabetes mellitus (GDM) is defined as abnormal glucose tolerance with onset or first recognition during pregnancy [1]. Worldwide, it affects approximately 2–20% of all pregnancies [2]. GDM has been shown to be associated with poor pregnancy outcome and substantial long-term adverse consequences for mothers and their offspring [3–7]. So far, the major risk factors related to GDM are older age at pregnancy, obesity, family history of T2DM and past history of GDM, previous poor obstetric history and genetics [8–12]. Insulin secretory defect accompanied by peripheral insulin resistance is an important characteristic of GDM [13]. Melatonin receptor 1B (MTNR1B) is an integral membrane protein that coupled to an inhibitory G protein and is expressed in pancreatic islets and pancreatic β cells [14–16]. It has been found that the increased expression of MTNR1B on β cells diminished intracellular cyclic cAMP levels, thereby inhibited the insulin secretion [17–19]. These findings suggested that MTNR1B may be involved in the development of GDM.

*MTNR1B* is located on human chromosome 11q21-q22, spanning about 22 kb and consisting of 3 exons and 1 intron. So far, 64 single-nucleotide polymorphisms (SNPs) have been validated in the *MTNR1B* gene (http://www.ncbi.nlm.nih.gov/SNP), and some of which were reported to be associated with GDM risk. The SNP rs10830963 is located in the unique intron between exon 1 (+5.6 kb) and exon 2 (−5.9 kb) of *MTNR1B* gene. Genotype–phenotype study of the SNP rs10830963 C>G showed that compared with the wild-type C allele of rs10830963, the variant G allele was associated with increased *MTNR1B* transcript levels in human islets [20]. The study of Li et al. indicated that G allele carrying genotype means a higher MTNR1B protein level, fasting blood glucose, fasting insulin and homeostasis model assessment for insulin resistance [21].

Because of the functional consequence of rs10830963 C>G, many association studies have examined its effect on the risk of GDM [22–35]. However, these studies presented inconsistent results. To clarify the effect of the *MTNR1B* rs10830963 C>G on the risk of GDM, we therefore performed a meta-analysis with a total of 5033 GDM patients and 5614 controls from 14 published case–control studies.

## Materials and methods

### Literature search strategy

We searched NCBI PubMed, Google Scholar and the Chinese National Knowledge Infrastructure (CNKI) databases for the association studies of *MTNR1B* rs10830963 polymorphism with the risk of GDM. The following key words ‘MTNR1B’ or ‘Melatonin receptor 1B’, ‘gestational diabetes mellitus’ and ‘variation’ or ‘polymorphism’ were used. The corresponding Chinese terms were used in the Chinese library. All studies were published up to March 1, 2019. In addition, we manually searched for additional published studies on this topic in the references cited in the retrieved studies.

The included studies in this meta-analysis had to meet the following criteria: evaluation of the *MTNR1B* rs10830963 C>G polymorphism and GDM risk; case–control study; genotype or allele distribution information in cases and controls for calculating odds ratio (OR) with corresponding 95% confidence interval (CI); the study was written in English or Chinese. Accordingly, family based studies, abstract, case reports, comments and reviews were excluded. If studies had overlapped subjects, only the largest study was included in the final analysis.

### Data extraction and quality score assessment

Two professional investigators (Huang B and Wang Y) independently reviewed the articles and extracted the data from eligible publications. The following information was extracted from each included study: first author, year of publication, country, diagnostic criteria, source of controls, number of cases and controls, genotype or allele distribution data of cases and controls, mean age, mean body mass index (BMI) and *P*-value of the Chi-square goodness of fit test for Hardy–Weinberg equilibrium (HWE) in controls.

In the present study, the Newcastle–Ottawa scale (NOS) scale was used to assess the quality of the eligible studies by Yu, X.Y. and Wang, Y.K. independently [36]. A score range of the scale was from 0 (the lowest) to 9 (the highest), and study with a score <5 were considered to be of low quality and those ≥5 to be of high quality. If there is disagreement in quality assessment, it can be resolved through discussion.

### Statistical analysis

Deviation of genotype frequencies of the *MTNR1B* rs10830963 C>G polymorphism in controls from HWE was tested by using the Chi-square goodness of fit test, and a *P*-value less than 0.05 was considered a departure from HWE. The odds ratio (OR) and 95% confidence interval (CI) were used to assess the strength of association between the *MTNR1B* rs10830963 C>G and GDM risk. The heterogeneity across the studies was assessed by the *Q* test, and was considered significant when a *P*-value less than 0.1 [37]. A fixed-effect model was used to calculate the pooled OR if the heterogeneity was not significant, otherwise, the random-effect model was adopted [38]. The potential source of heterogeneity across the included studies was explored with meta-regression analyses by ethnicity (Asian and Caucasian), diagnostic criteria(ADA and others) and assessed literature quality (high and low). Both Begg’s and Egger’s tests were used to test for publication bias [39,40]. A *P*-value < 0.05 was considered as an indication for the potential presence of publication bias. Sensitivity analyses were done to assess the influence of individual study on the pooled ORs. All analyses were performed by using Stata software, version 12.0 (Stata Corp LP, College Station, TX, U.S.A.). In addition, false positive report probability (FPRP) was estimated to assess the robustness of foundings statistically significant association by using the method described by Wacholder et al. [41]. The FPRP threshold was set to 0.2, and the prior probability was set to 0.1 to detect the noteworthiness for OR of 1.5 or 0.67, with an alpha level equal to the observed *P*-value. An FPRP less than 0.2 was considered as a noteworthy association [41,42].

### Trial sequential analysis (TSA)

Meta-analysis might be affected by type I error due to the increased risk of random error and repeated significance testing [43]. Trial sequential analysis (TSA) was used to reduce random errors and increase the robustness of the conclusions by estimating the amount of required information size (RIS) and the threshold for statistical significance [44]. A 5% significance level for type I errors, 20% significance level for type II errors (80% power) and 20% relative risk reduction (RRR) were defined and a TSA monitoring boundary were determined. TSA was conducted in allelic model and positive results of meta-analysis were tested. When the cumulative *Z*-curve crosses the TSA boundary or enters the insignificance area, a sufficient level of evidence has been reached, and no further studies are necessary. However, when the *Z-*curve does not exceed any of the boundaries and the required sample size has not been reached, evidence to reach a conclusion is insufficient [45]. Review Manager (RevMan) version 5.2 and TSA version 0.9.5.10 beta softwares were used in data processing.

## Results

### Characteristics of included studies

**Figure 1:**
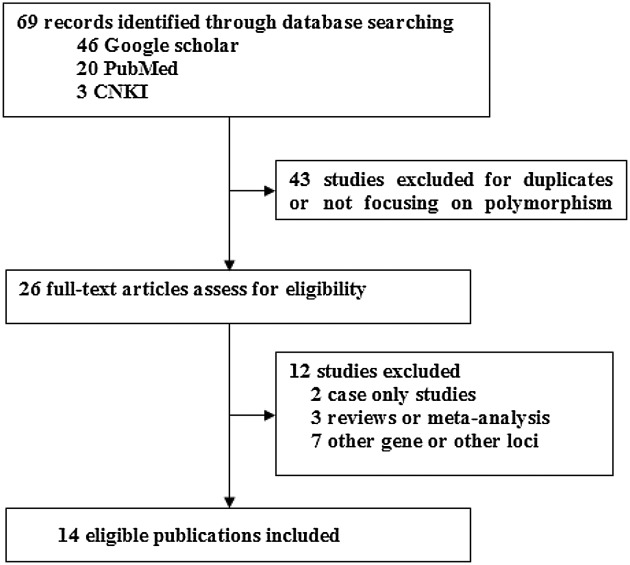
Flowchart of the process of identification of eligible studies

The flowchart of study selection for this meta-analysis is presented in Figure 1. A total of 69 studies were found using our literature search strategy, of which 43 studies were excluded because of duplicates or not on the topic of polymorphisms and GDM risk. After full-text reviews of the remaining 26 articles, 12 studies were excluded for the following reasons: 2 studies were case only studies, 3 studies were review or meta-analysis articles, 7 studies didn’t focus on the topic of the *MTNR1B* rs10830963 C>G and GDM risk. Finally, 14 studies with 5033 GDM patients and 5614 controls were selected in present study. According to the evaluation of NOS scale, 12 of the included literatures were considered to be of high quality (score ≥ 5) and 2 of them were of low quality (score < 5). Main characteristics of the included studies are shown in Table 1. The genotype frequency distributions of the rs10830963 C>G in controls were in agreement with HWE in all included studies except for the two by Vlassi et al. [25] and Liu et al. [32].

**Table 1:** Characteristics of the studies included in the meta-analysis

Author, year	Country	Diagnostic criteria	Genotyping methods	Controls	No. of case/ control	MAF case/ control	Mean age of cases/controls	Mean BMI of cases/controls	*p*_HWE_ for controls	NOS score
Deng, Z., 2011	China	ADA	Sequencing	NGT	87/91	0.52/0.41	31.8 ± 4.6/29.7 ± 3.5	23.6 ± 3.0/21.5 ± 2.4	0.84	4
Kim, J.Y., 2011	Korea	ADA	TaqMan	NGT	908/966	0.52/0.45	33.1/32.2	23.3 ± 4.0/21.4 ± 2.9	0.53	7
Wang, Y., 2011	China	ADA	TaqMan	NGT	700/1029	0.46/0.43	30.0/32.0	21.5/21.7	0.81	8
VlassiM, 2012	Greece	ADA	PCR-RFLP	NGT	77/98	0.41/0.28	35.4 ± 4.4/31.3 ± 5.2	25.8 ± 5.1/26.7 ± 6.2	0.02	4
HuopioH, 2013	Finland	ADA	Sequenom Assay/TaqMan	NGT	533/407	0.47/0.35	32.6/29.9	26.3 ± 4.7/24.1 ± 3.8	0.98	8
Li. C., 2013	China	IADPSG	PCR-RFLP	NGT	350/480	0.45/0.40	32.4 ± 4.8/31.9 ± 5.2	25.3 ± 5.2/24.6 ± 4.6	0.79	8
Qi, J., 2013	China	IADPSG	Sequencing	NGT	110/110	0.54/0.44	28.7 ± 3.1/28.1 ± 2.4	NA/NA	0.43	6
Vejrazkova, D., 2014	Czech	WHO	TaqMan	NGT	458/422	0.38/0.29	34.1 ± 6.1/34.8 ± 15.1	24.3 ± 4.9/23.7 ± 4.2	0.48	8
Wang, X., 2014	China	ADA	PCR-RFLP	NGT	184/235	0.42/0.45	28.2 ± 3.8/27.9 ± 4.1	21.2 ± 1.8/20.7 ± 1.4	0.53	6
Junior, J.P., 2015	Brazil	ADA	Real-time PCR	Healthy pregnant	183/183	0.28/0.20	32/29	32.0/25.4	0.11	7
Liu, Q., 2015	China	ADA	TaqMan	NGT	674/674	0.51/0.44	31.6/32.1	24.4/25.2	0.02	8
Tarnowski, M., 2017	Poland	IADPSG	TaqMan	NGT	204/207	0.39/0.31	31.7 ± 4.5/29.2 ± 5.0	25.1 ± 5.5/23.0 ± 4.0	0.112	7
Popova, P.V., 2017	Russia	ADA	RT-PCR	Healthy pregnant	278/179	0.35/0.31	31.8 ± 4.8/29.4 ± 4.8	25.7 ± 5.9/22.9 ± 4.5	0.426	6
Rosta, K., 2017	Hungary and Austria	IADPSG	KASP assay	—	287/533	0.33/0.30	—	—	0.975	5

Note: ADA, American Diabetes Association; IADPSG, International Association of the Diabetes and Pregnancy Study Groups; NGT, Normal Glucose Tolerance; MAF, Minor Allele Frequency; BMI, Body Mass Index; HWE, Hardy–Weinberg Equilibrium; NOS, Newcastle–Ottawa Scale.

### Association between the *MTNR1B* rs10830963 C>G and GDM risk

**Figure 2:**
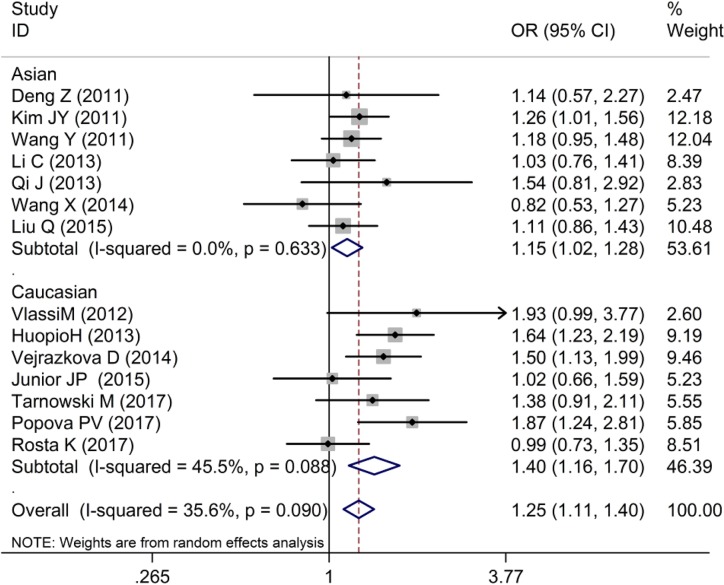
Forest plot on the risk of GDM associated with rs10830963 (CG vs. CC)

**Figure 3:**
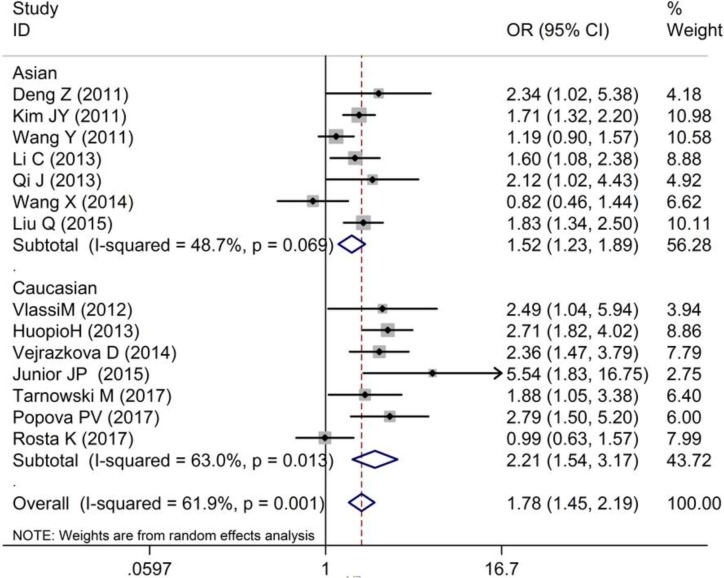
Forest plot on the risk of GDM associated with rs10830963 (GG vs. CC)

**Figure 4:**
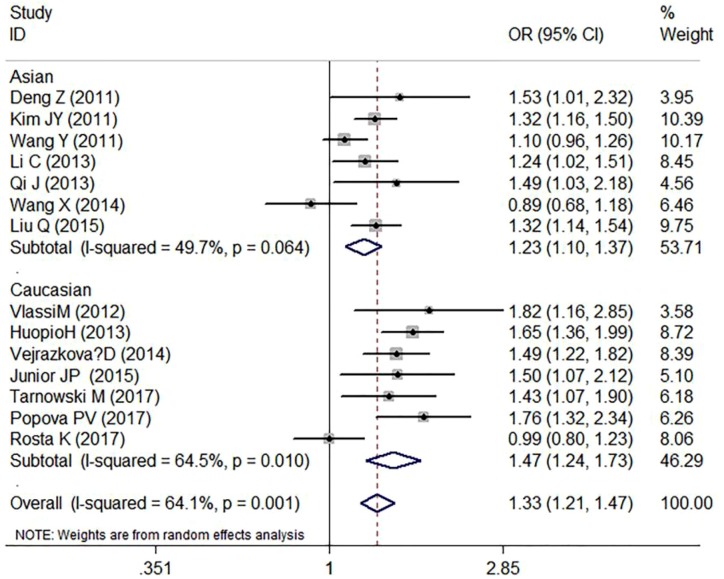
Forest plot on the risk of GDM associated with rs10830963 (G vs. C)

As shown in Table 2 and Figures 2–4, we found that the variant G allele of rs10830963 polymorphism was significantly associated with an increased risk of GDM (CG vs. CC: OR = 1.25, 95% CI = 1.11−1.40, *P* < 0.001, *P*_heterogeneity_ = 0.090, *I^2^* = 35.6%; GG vs. CC: OR = 1.78, 95% CI = 1.45−2.19, *P* < 0.001, *P*_heterogeneity_ = 0.001, *I^2^* = 61.9%. G vs. C: OR = 1.33, 95% CI = 1.21−1.47, *P* < 0.001, *P*_heterogeneity_ = 0.001, *I^2^* = 64.1%), respectively.

**Table 2:** Meta-analysis of the *MTNR1B* rs10830963 polymorphism on GDM risk

Subgroup	Heterozygous (CG vs. CC)				Homozygous (GG vs. CC)				Allele mogel (G vs. C)			
	No. of studies	Case/ Control	OR (95% CI)	*P*_Effect_	No. of studies	Case/ Control	OR (95% CI)	*P*_Effect_	No. of studies	Case/Control	OR (95% CI)	*P*_Effect_
Overall	14	3952/4736	1.25 (1.11–1.40)	<0.001	14	2628/2966	1.78 (1.45–2.19)	<0.001	14	10066/11228	1.33 (1.21–1.47)	<0.001
Ethnicity												
Asian	7	2271/2916	1.15 (1.02–1.28)	0.020	7	1543/1796	1.52 (1.23–1.89)	<0.001	7	6026/7170	1.23 (1.10–1.37)	<0.001
Caucasian	7	1681/1820	1.40 (1.16–1.70)	<0.001	7	1085/1170	2.21 (1.54–3.17)	<0.001	7	4040/4058	1.47 (1.24–1.73)	<0.001

In the stratified analysis by ethnicity, as shown in Table 2 and Figures 2–4, we found that rs10830963 polymorphism was significantly associated with a relatively higher GDM risk in the above three models in Asians (CG vs. CC: OR = 1.15, 95% CI = 1.02−1.28, *P* = 0.020, *P*_heterogeneity_ = 0.633, *I^2^* = 0.0%; GG vs. CC: OR = 1.52, 95% CI = 1.23−1.89, *P* < 0.001, *P*_heterogeneity_ = 0.069, *I^2^* = 48.7%; G vs. C: OR = 1.23, 95% CI = 1.10−1.37, *P* < 0.001, *P*_heterogeneity_ = 0.064, *I^2^* = 49.7%) and in Caucasians (CG vs. CC: OR = 1.40, 95% CI = 1.16−1.70, *P* < 0.001, *P*_heterogeneity_ = 0.088, *I^2^* = 45.5%; GG vs. CC: OR = 2.21, 95% CI = 1.54−3.17, *P* < 0.001, *P*_heterogeneity_ = 0.013, *I^2^* = 63.0%; G vs. C: OR = 1.47, 95% CI = 1.24−1.73, *P* < 0.001, *P*_heterogeneity_ = 0.010, *I^2^* = 64.5%).

### Evaluation of heterogeneity

In this present study, the *Q* test was used to evaluate the heterogeneity across the included studies and the heterogeneity across studies was found in most of comparisons. As shown in Table 2, the *Q* test suggested that a low to high heterogeneity across studies presented in most of comparisons. We then used the meta-regression analysis to explore the source of heterogeneity by ethnicity (Asian and Caucasian), diagnostic criteria (ADA and others) and assessed literature quality (score ≥ 5 and <5), and found that they didn’t contribute to the main observed heterogeneity across the studies, effect of ethnicity (CG vs. CC: *t* = −2.05, *P* = 0.063; GG vs. CC: *t* = −1.78, *P* = 0.101; G vs. C: *t* = 1.91, *P* = 0.080), diagnostic criteria (CG vs. CC: *t* = 1.03, *P* = 0.325; GG vs. CC: *t* = 1.41, *P* = 0.183; G vs. C: *t* = 1.26, *P* = 0.230) and literature quality (CG vs. CC: *t* = −0.98, *P* = 0.344; GG vs. CC: *t* = −0.22, *P* = 0.826; G vs. C: *t* = −0.10, *P* = 0.923).

### Sensitivity analyses

**Figure 5:**
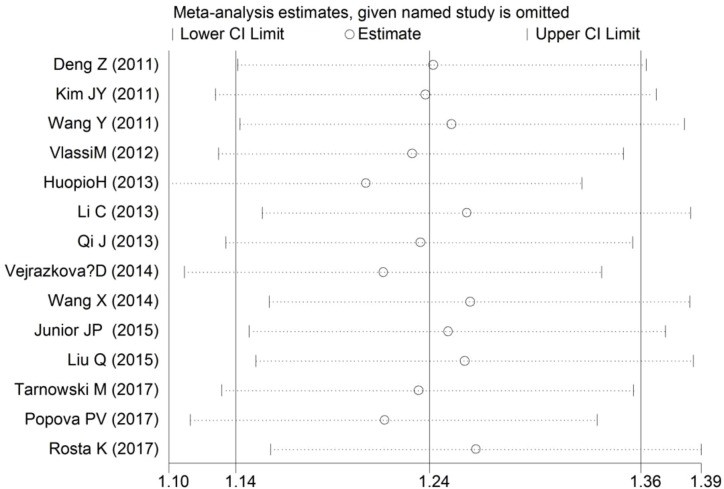
Sensitivity analyses of the association between rs10830963 C>G and GDM risk under the CG vs. CC comparison

**Figure 6:**
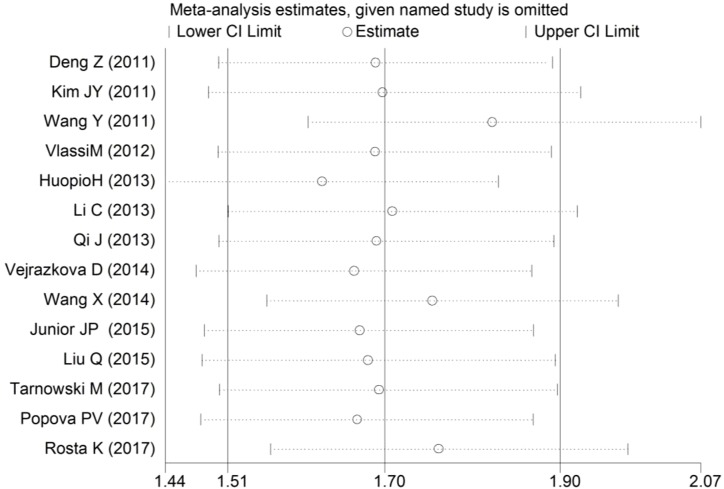
Sensitivity analyses of the association between rs10830963 C>G and GDM risk under the GG vs. CC comparison

Sensitivity analyses of the association between the *MTNR1B* rs10830963 C>G polymorphism and GDM risk were performed to assess the stability of the pooled ORs under the CG vs. CC and GG vs. CC comparisons. The leave-one-out analysis showed that no single study dramatically influenced the pooled ORs (Figures 5 and 6).

### Publication bias

**Figure 7:**
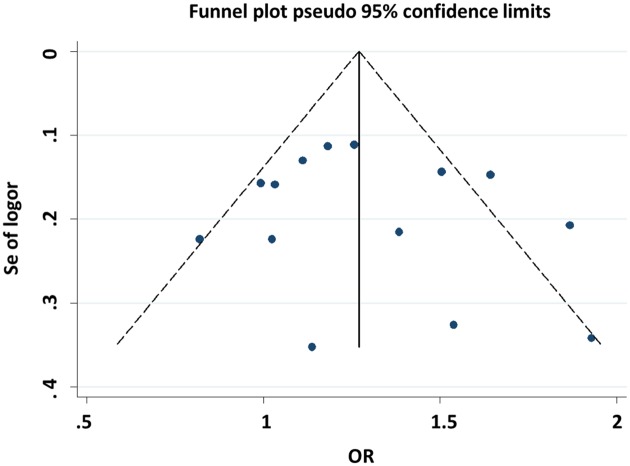
Begg’s funnel plot for publication bias test (CG vs. CC)

**Figure 8:**
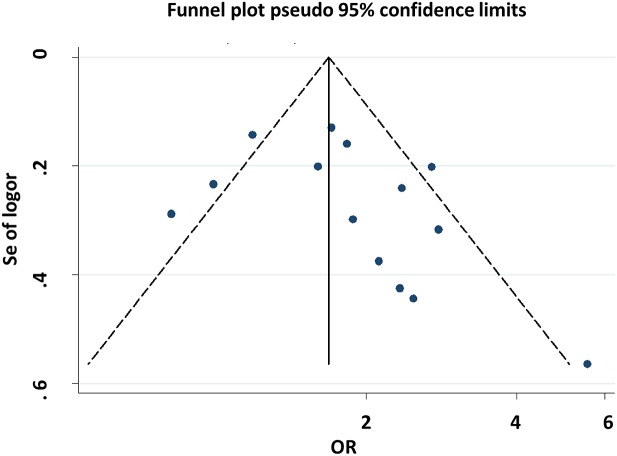
Begg’s funnel plot for publication bias test (GG vs. CC)

Both Begg’s and Egger’s tests were performed to evaluate the publication bias of the included studies. The shape of the funnel plots did not reveal any evidence of obvious asymmetry for all genetic models (Figures 7 and 8), and the Begg’s and Egger’s tests did not present any significantly statistical evidence of publication bias for any of the genetic models in the overall meta-analysis (CG vs. CC: *P*_Begg’s_ = 0.913 and *P*_Egger’s_ = 0.655, GG vs. CC: *P*_Begg’s_ = 0.063 and *P*_Egger’s_ = 0.186).

### FPRP analysis results

FPRP was adopted to assess the noteworthiness of the significant associations between the *MTNR1B* rs10830963 C>G polymorphism and GDM risk. At the prior probability of 0.1 and FPRP cut-off value of 0.2, the FPRP values for the significant findings in the heterozygous genotype comparison (CG vs. CC), homozygote model (GG vs. CC) and allele model (G vs. C) of overall were 0.005, 0.008 and 0.004, respectively. Moreover, the FPRP values for the significant findings in the studied three models were 0.153, 0.009 and 0.010 in Asians, 0.007, 0.047 and 0.006 in Caucasians, respectively. As shown in Table 3.

**Table 3:** FPRP analysis for the significant associations of the *MTNR1B* rs10830963 C>G polymorphism and GDM risk

	OR (95% CI)	Prior probability					
		0.25	0.1	0.01	0.001	0.0001	0.00001
**Overall**							
CG vs. CC	1.25 (1.11–1.40)	0.002	0.005	0.056	0.375	0.857	0.984
GG vs. CC	1.78 (1.45–2.19)	0.003	0.008	0.083	0.477	0.901	0.989
G vs. C	1.33 (1.21–1.47)	0.001	0.004	0.038	0.286	0.800	0.976
**Asian**							
CG vs. CC	1.15 (1.02–1.28)	0.057	0.153	0.664	0.952	0.995	1.000
GG vs. CC	1.52 (1.23–1.89)	0.003	0.009	0.092	0.506	0.911	0.990
G vs. C	1.23 (1.10–1.37)	0.003	0.010	0.097	0.519	0.915	0.991
**Caucasian**							
CG vs. CC	1.40 (1.16–1.70)	0.002	0.007	0.074	0.446	0.889	0.988
GG vs. CC	2.21 (1.54–3.17)	0.016	0.047	0.351	0.845	0.982	0.998
G vs. C	1.47 (1.24–1.73)	0.002	0.006	0.060	0.393	0.866	0.985

### Trial sequential analysis (TSA)

**Figure 9:**
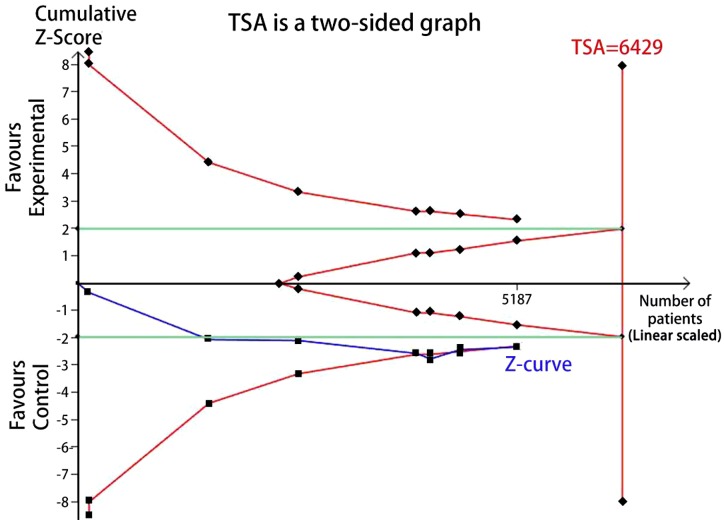
TSA for rs10830963 under the heterozygote model among Asians (CG vs. CC)

**Figure 10:**
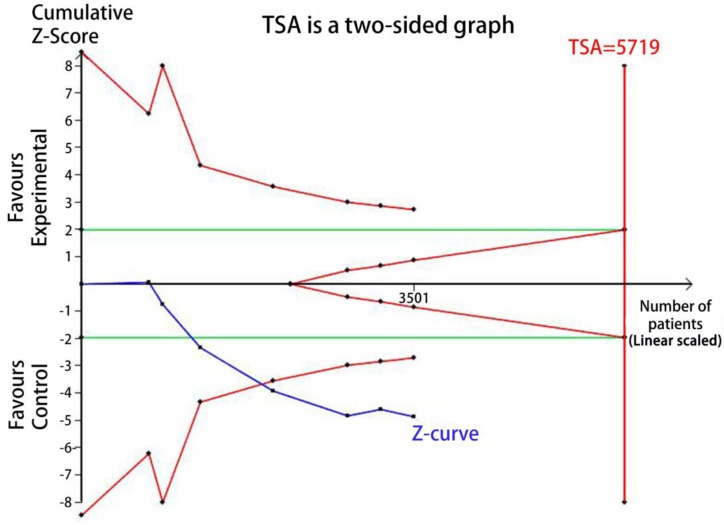
TSA for rs10830963 under the heterozygote model among Caucasians (CG vs. CC)

**Figure 11:**
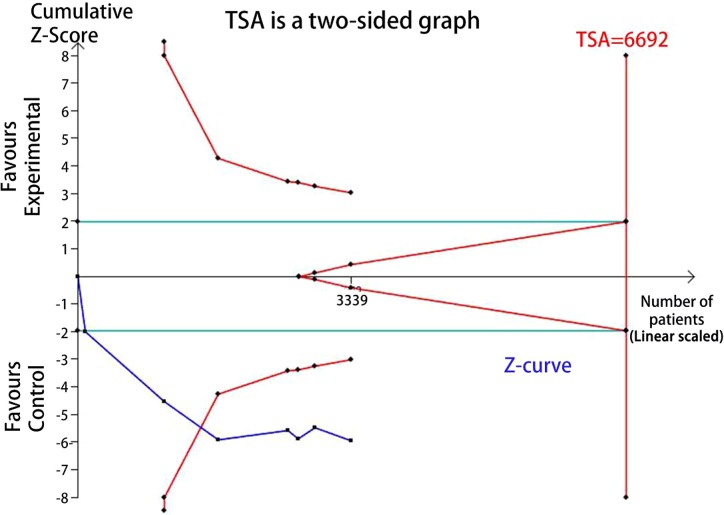
TSA for rs10830963 under the homozygote model among Caucasians (GG vs.CC)

**Figure 12:**
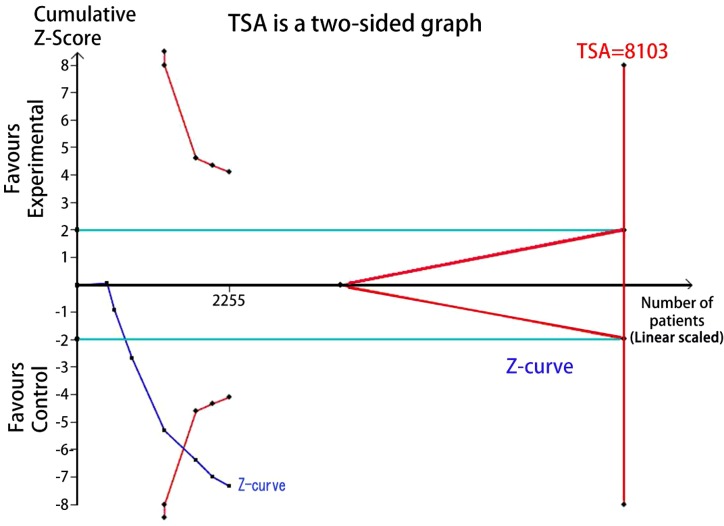
TSA for rs10830963 under the homozygote model among Caucasians (GG vs. CC)

TSA was performed to reduce the random errors and increase the robustness of the conclusions. The TSA of the heterozygote (CG vs. CC) and homozygote models (GG vs. CC) for rs10830963 C>G among Asians and Caucasians (Figures 9–12), indicated that the cumulative Z-curve crossed both the conventional cut-off value and the TSA boundaries, and confirmed that the SNP was mainly associated with susceptibility to GDM. Even if the cumulative amount information didn’t reach the required information size (RIS), the results of TSA suggested that no further study evidence was needed to verify the conclusion.

## Discussion

Gestational diabetes mellitus (GDM) is considered as an early form of diabetes, which might increase the risk of adverse pregnancy outcomes and substantial long-term adverse health among mothers and their offspring. Pregnant women with GDM are at a relatively high risk of developing Type 2 diabetes in the future [46]. Moreover, GDM also can be increase the hypertension, obesity, dyslipidemia and cardiovascular disease [4–6,47]. Therefore, it is urgent to clarify the etiology and pathogenesis of GDM.

A number of candidate gene-based association studies and genome-wide association studies (GWAS) have shown that some GDM relation genetic variants could provide insight into pathogenetic mechanisms underlying the disease [48–50]. Functional studies have shown that these diabetogenic genes took part in the process of developing GDM by impairing β-cell function, insulin resistance or abnormal utilization of glucose etc [48,49]. Our present study showed that the frequency of *MTNR1B* rs10830963 G allele was relatively higher in GDM cases than that in controls, suggesting a significant association with an increased GDM risk.

GDM could occur when the pancreatic islet β cells were impaired by an increased insulin resistance during pregnancy [51]. MTNR1B is a G-protein coupled 7-transmembrane receptor and could influence pancreatic β-cell function and fasting plasma glucose (FPG) level [14,15]. Studies have shown that genetic variations of MTNR1B gene are associated with insulin secretion and impaired β-cell function. A study of genetics and quantitative traits analysis of Palmer et al*.* revealed a significant association between *MTNR1B* and the glucose disposition index and acute insulin response among T2DM [52]. Meanwhile, experimental studies have confirmed that comparing with the wild-type C allele of rs10830963, the variant G allele caused an increased expression of *MTNR1B* and to be related to the risk of T2DM or GDM [52–54]. Our study showed that *MTNR1B* rs10830963 C>G might possibly increase the incidence of GDM. This may due to the observation that the increased expression of MTNR1B on β cells diminished intracellular cyclic cAMP level, thereby inhibited the glucose-stimulated insulin secretion.

A previous meta-analysis by Zhang et al*.* observed a statistically significant association between the *MTNR1B* variant CG/GG genotype and GDM risk (OR = 1.24, 95% CI = 1.14−1.35) [55]. However, this meta-analysis only included 5 studies (4 studies for Asians and 1 for Caucasians) with 2122 GDM patients and 2664 control subjects, and was unable to do the subgroup analysis by ethnicity to reveal the effect of *MTNR1B* rs10830963 C>G polymorphism on GDM risk in different ethnic populations.We summarized the evidence to date with 5033 GDM patients and 5614 controls by a meta-analysis with TSA, and found that compared with the wild CC genotype, the variant CG and GG genotype were significantly associated with an increased risk of GDM, respectively. Furthermore, the subgroup analysis by ethnicity revealed that rs10830963 C>G polymorphism was significantly associated with the risk of GDM both in Asians and in Caucasians. Obvious heterogeneity across studies was observed in data processing in this meta-analysis. We then used the meta-regression analysis to explore the potential source of heterogeneity across the studies by ethnicity, diagnostic criteria and assessed literature quality, and found that they didn’t contribute to the main observed heterogeneity.

FPRP analysis is an efective approach to verify the noteworthiness of signifcant association fndings. In the present study, we performed a relatively stringent FPRP threshold of 0.2. We found that the FPRP values of the observed signifcant associations between *MTNR1B* rs10830963 C>G and GDM risk was much lower than the preset threshold. It suggests that the positive findings both in overall analysis and racial-related subgroup analysis of heterozygous and homozygous models are probability authentic and reliable. Hence, we believe the association of rs10830963 C>G and GDM risk are credible to some extent. Further, a TSA indicated that the cumulative *Z*-curve crossed the conventional cut-off value and the TSA boundaries both in Aisan and Caucasian subgroup analyzes and confirmed that the rs10830963 SNP was mainly associated with susceptibility to GDM. The results of TSA suggesting that no additional researches are required to further evaluate the findings.

The current meta-analysis more comprehensively makes the relationship clear between *MTNR1B* rs10830963 and GDM risk. However, some limitations should be point out in the current meta-analysis. First, although the Begg’s and Egger’s tests did not detect any significantly statistical evidence of publication bias, selection bias could exist because only published case–control studies were included. Second, the small sample size might limit the statistical power of the study. Third, the findings of present study were based on the unadjusted results. Due to lack of individual-level data prevented us from making further analysis to identify any genotype–environment interaction between rs10830963 C>G and metabolic traits, such as FPG, pancreatic β-cell function, acute insulin response or indices for insulin sensitivity.

## Conclusion

In summary, the current meta-analysis with TSA indicates that the variant G allele of *MTNR1B* rs10830963 C>G polymorphism significantly increases the risk of GDM. Further functional experimental studies are warranted to help explore and clarify the potential mechanism.

## Abbreviations

CI: confidence interval
FPG: fasting plasma glucose
FPRP: false positive report probability
GDM: gestational diabetes mellitus
HWE: Hardy–Weinberg equilibrium
MTNR1B: melatonin receptor 1B
OR: odds ratio
RIS: required information size
TSA: trial sequential analysis
