# Correction: A functional polymorphism rs10830963 in melatonin receptor 1B associated with the risk of gestational diabetes mellitus

**DOI:** 10.1042/BSR-20190744_COR

**Published:** 2020-02-28

**Keywords:** Gestational diabetes mellitus, MTNR1B, Polymorphism, meta analysis, Trial sequential analysis

The authors of the original article A functional polymorphism rs10830963 in melatonin receptor 1B associated with the risk of gestational diabetes mellitus (*Bioscience Reports* 39(12) https://doi.org/10.1042/BSR20190744) would like to correct several data calculation points. In the original article, they had made a mistake in extracting the minimum allele frequency (MAF) of rs10830963 polymorphism from one included study [1]. The frequencies of rs10830963G allele 0.36 in the GDM group and 0.28 in the control group were therefore incorrectly calculated as 0.33 and 0.30, respectively. After recalculation using the corrected frequencies, and the association between the rs10830963 (Comparisons: CG vs. CC, GG vs. CC and G vs. C), the variant rs10830963 G allele was still significantly associated with an increased GDM risk (CG vs. CC: OR = 1.44, 95%CI = 1.06 -1.95; GG vs. CC: OR = 2.06, 95% CI = 1.26 - 3.37; G vs. C: OR = 1.44, 95% CI = 1.16 - 1.78). The change in data calculation therefore did not affect the final conclusion of their study.

Figures and tables have been updated in order to reflect this, and can be found in their correspondence piece [2].
